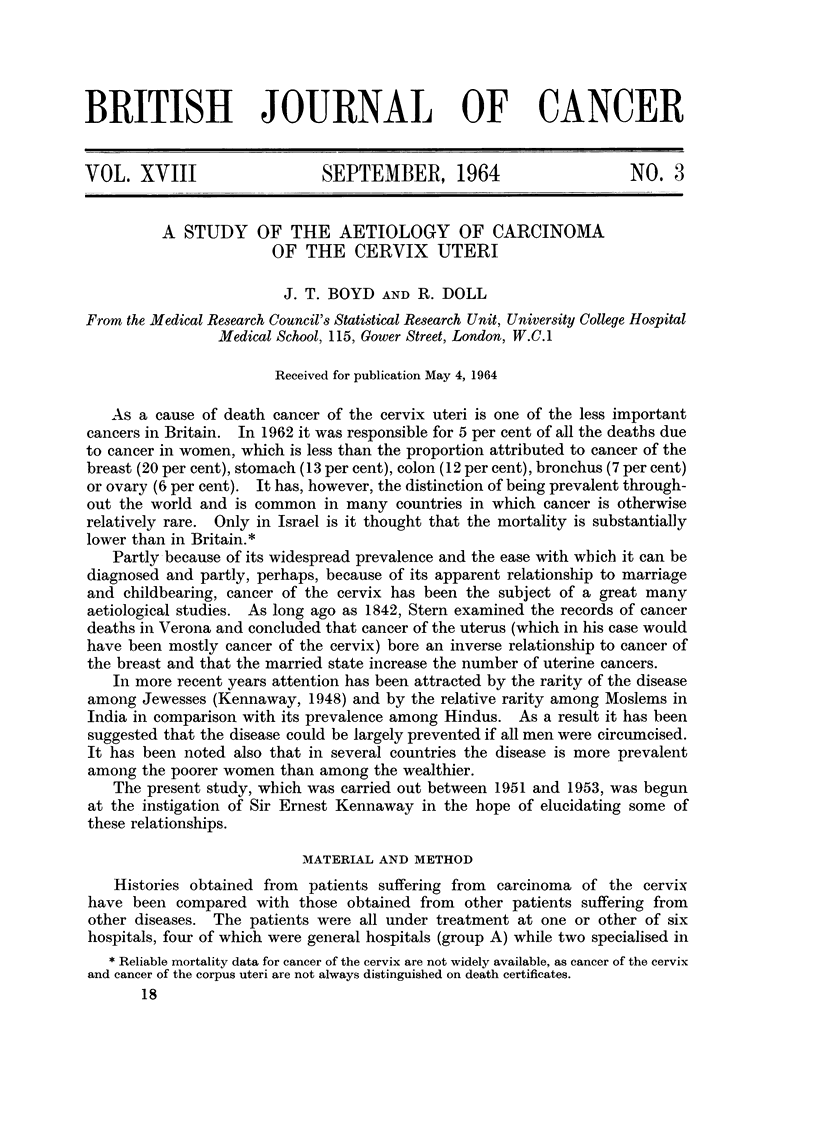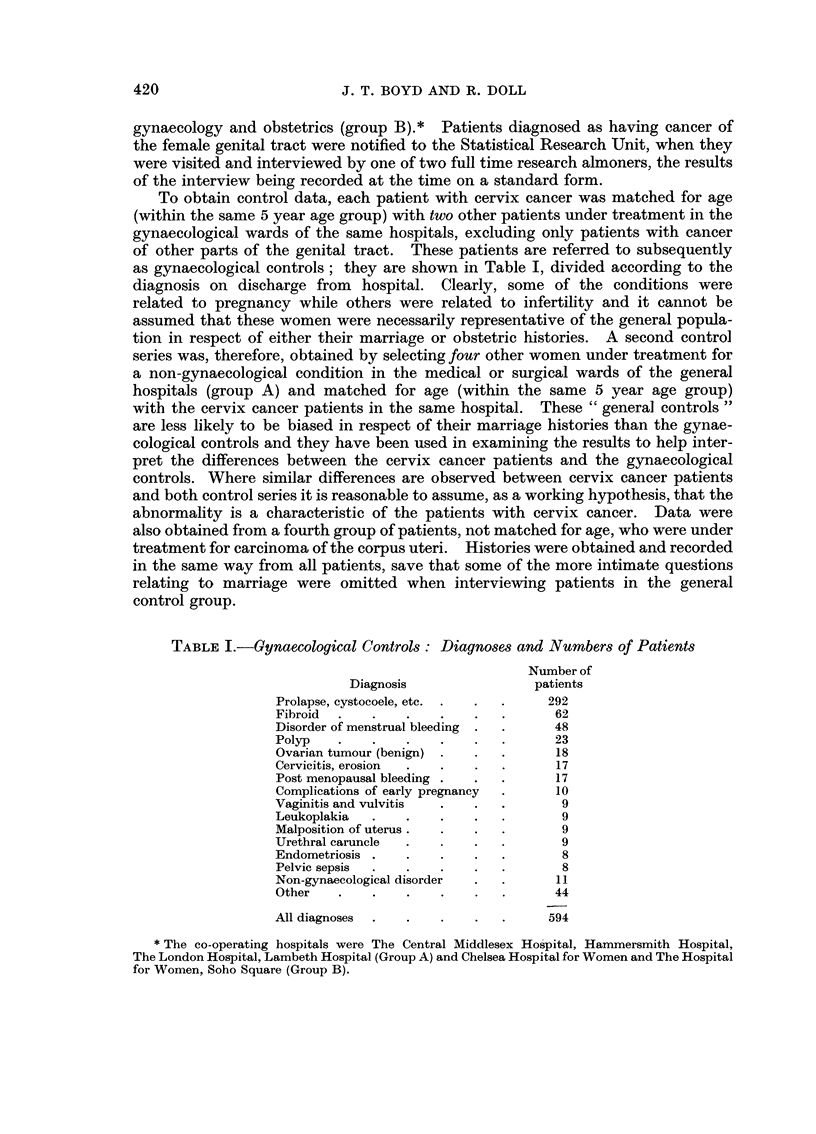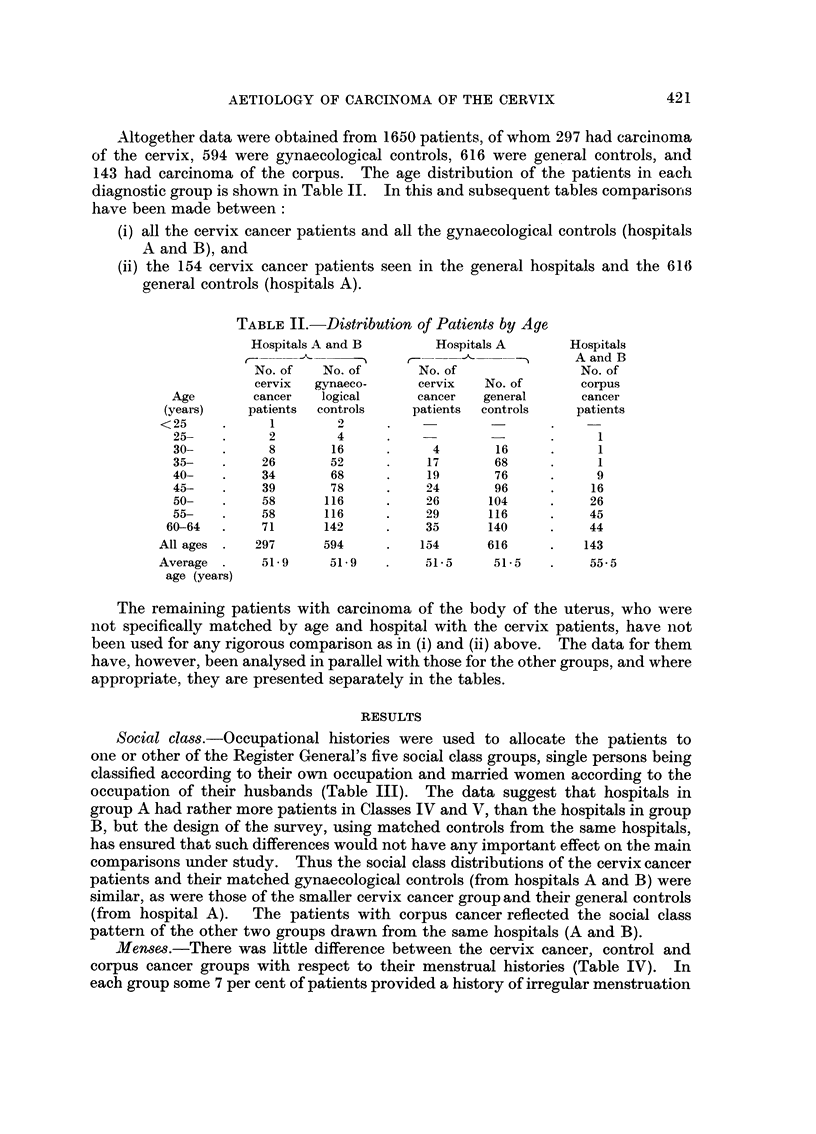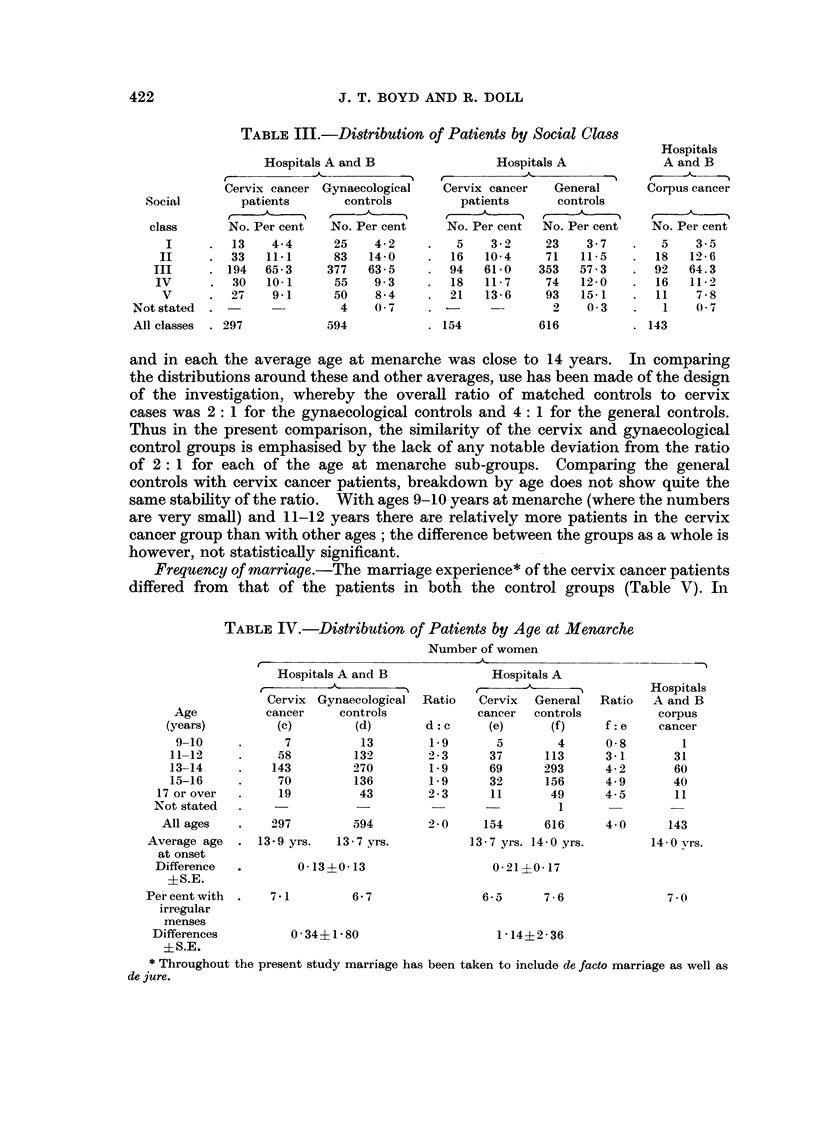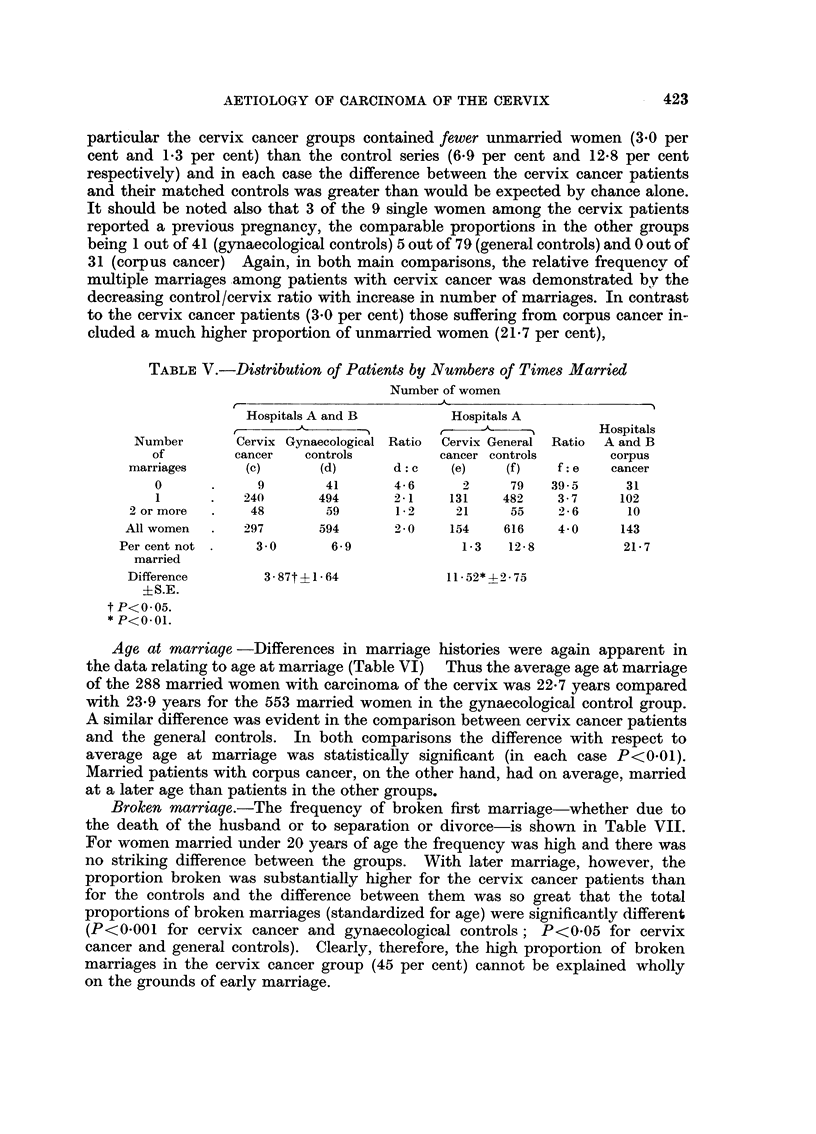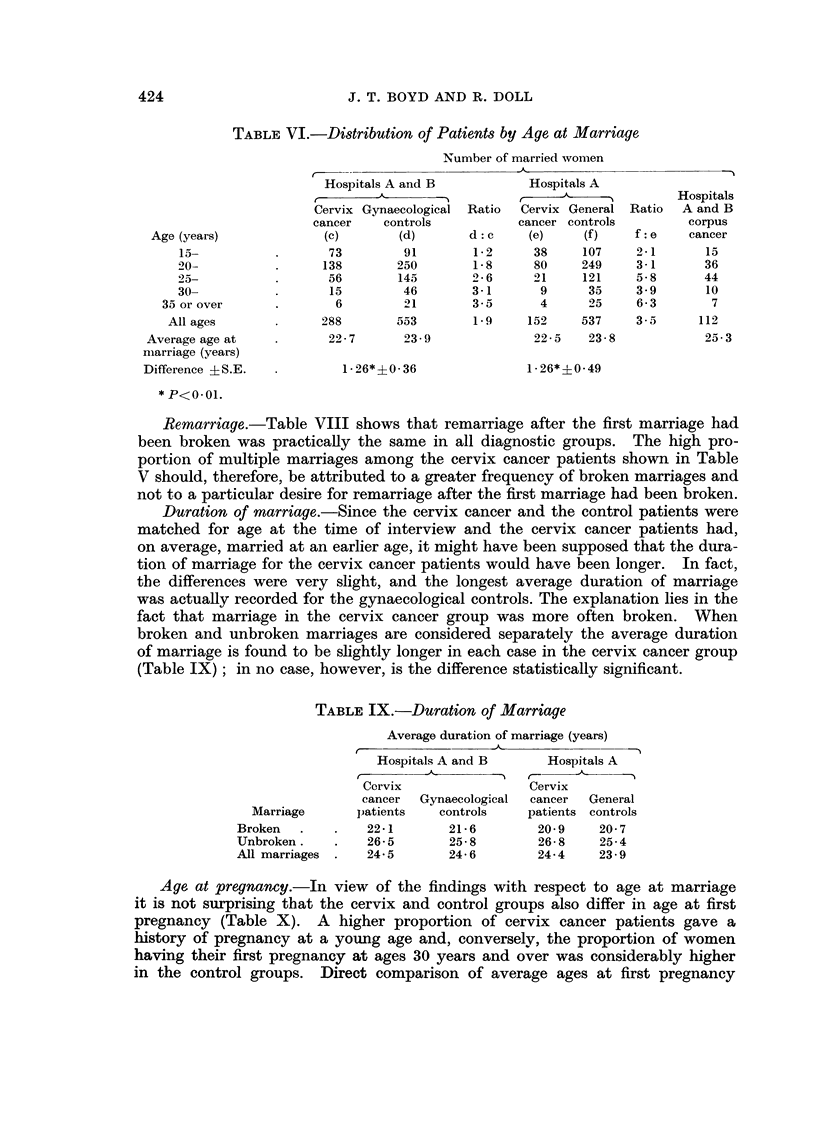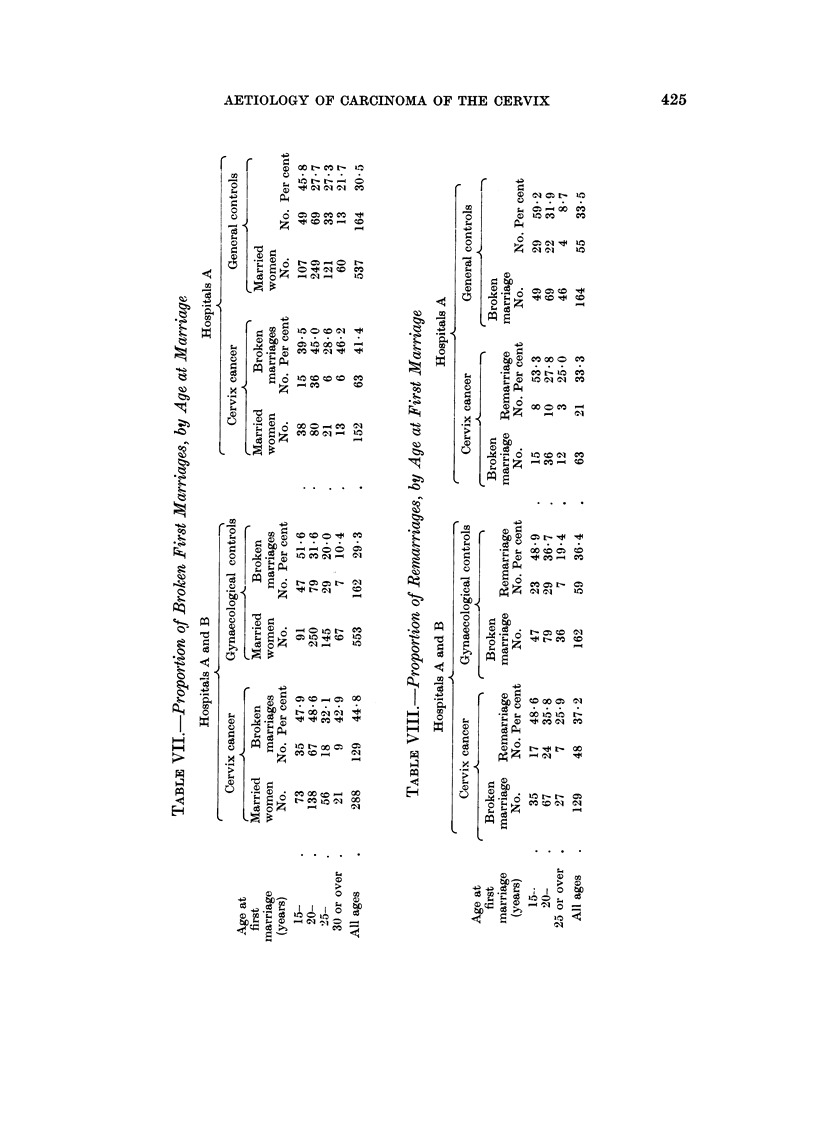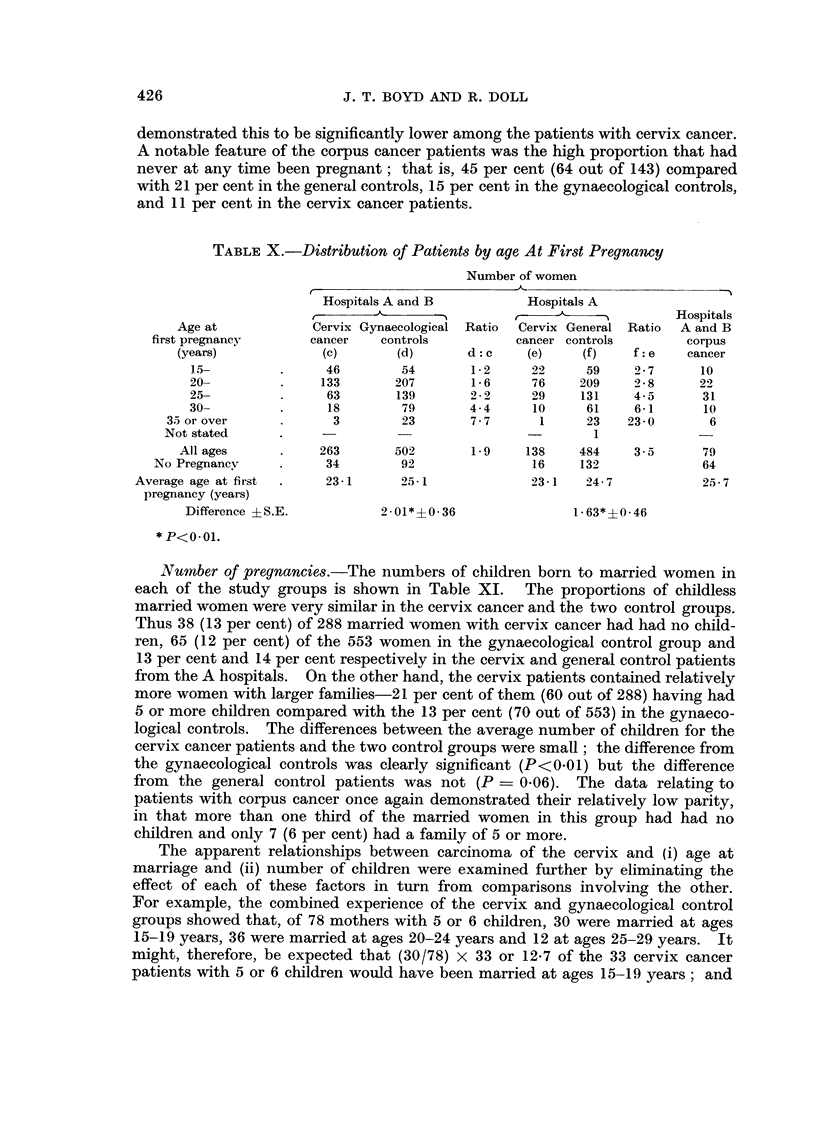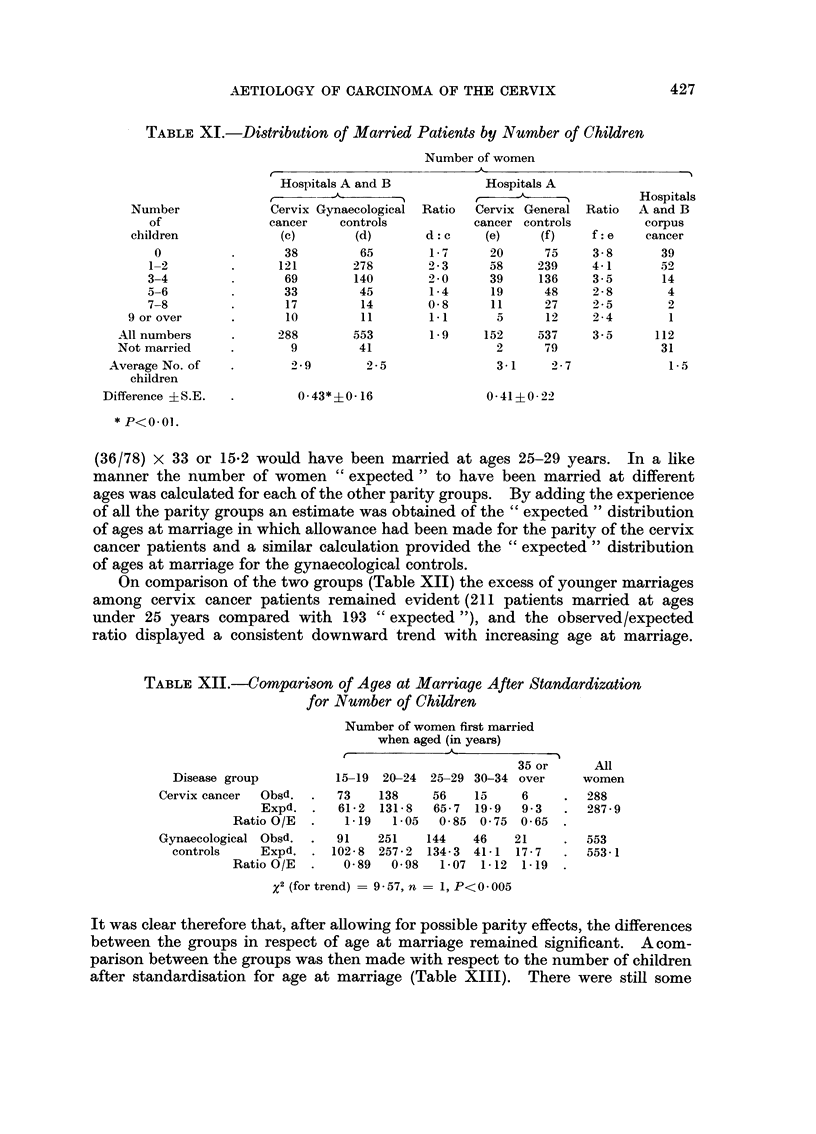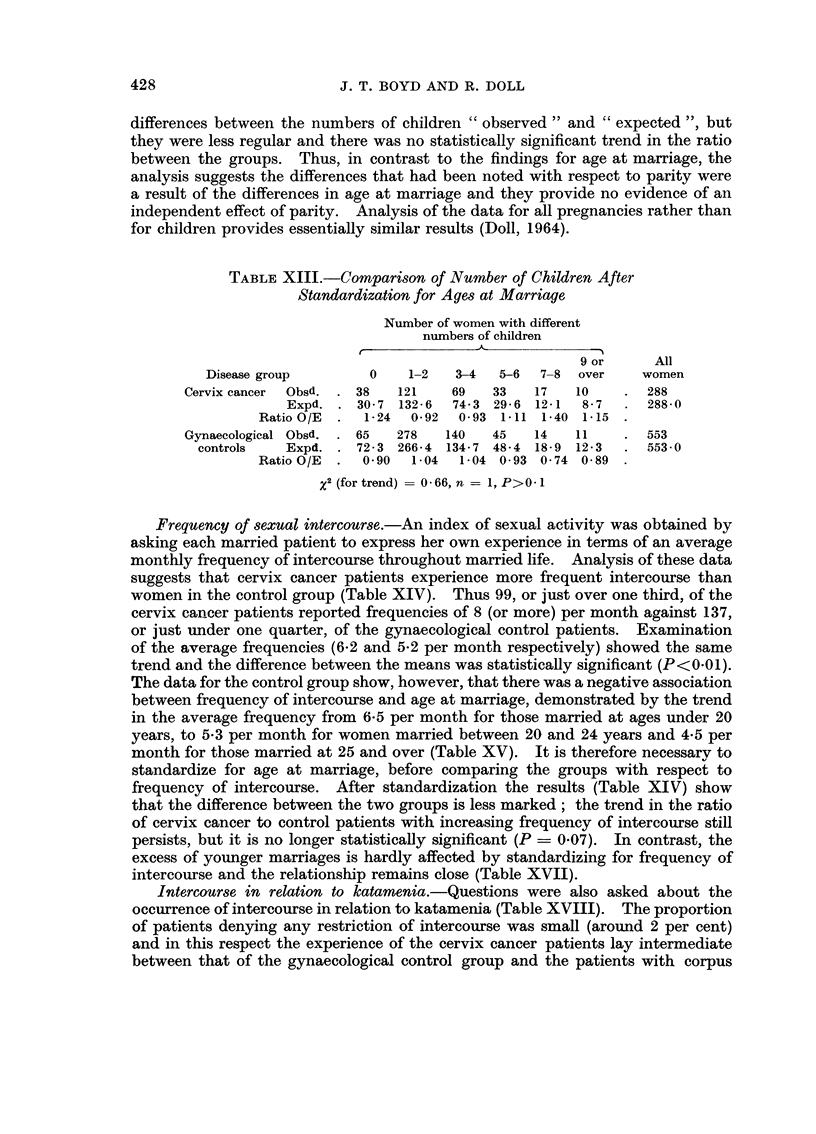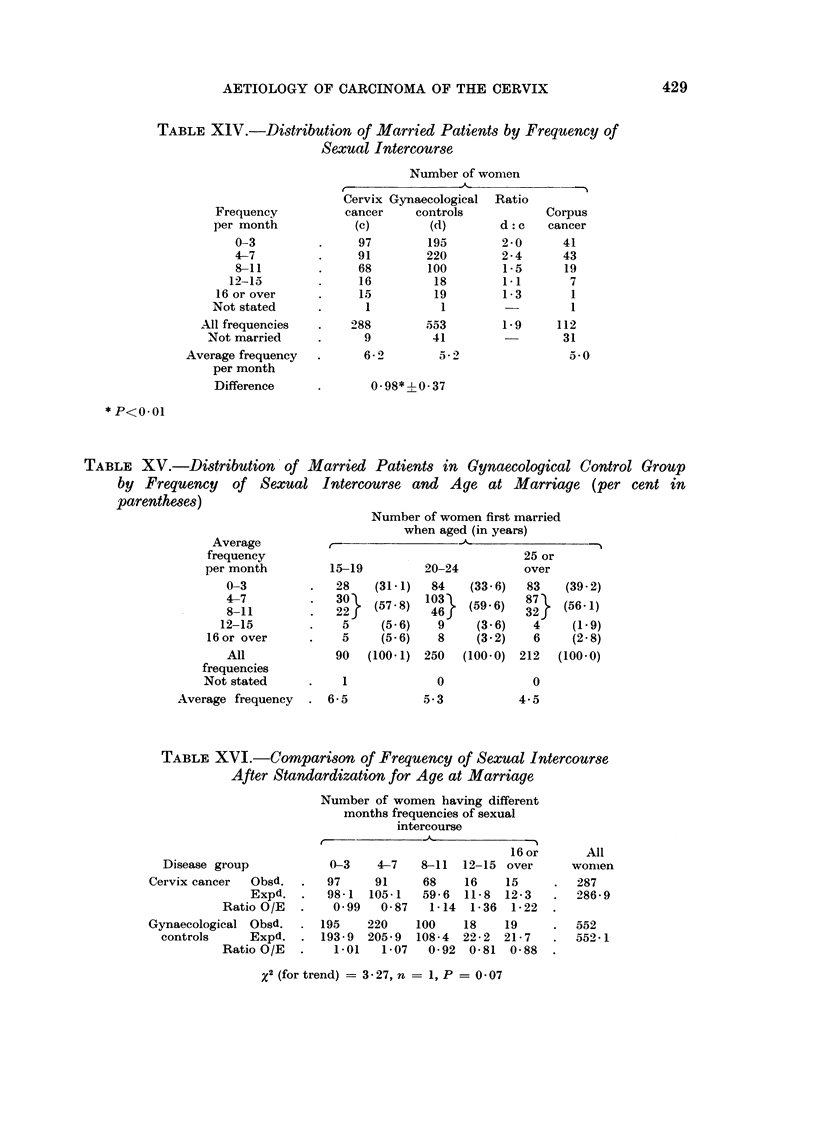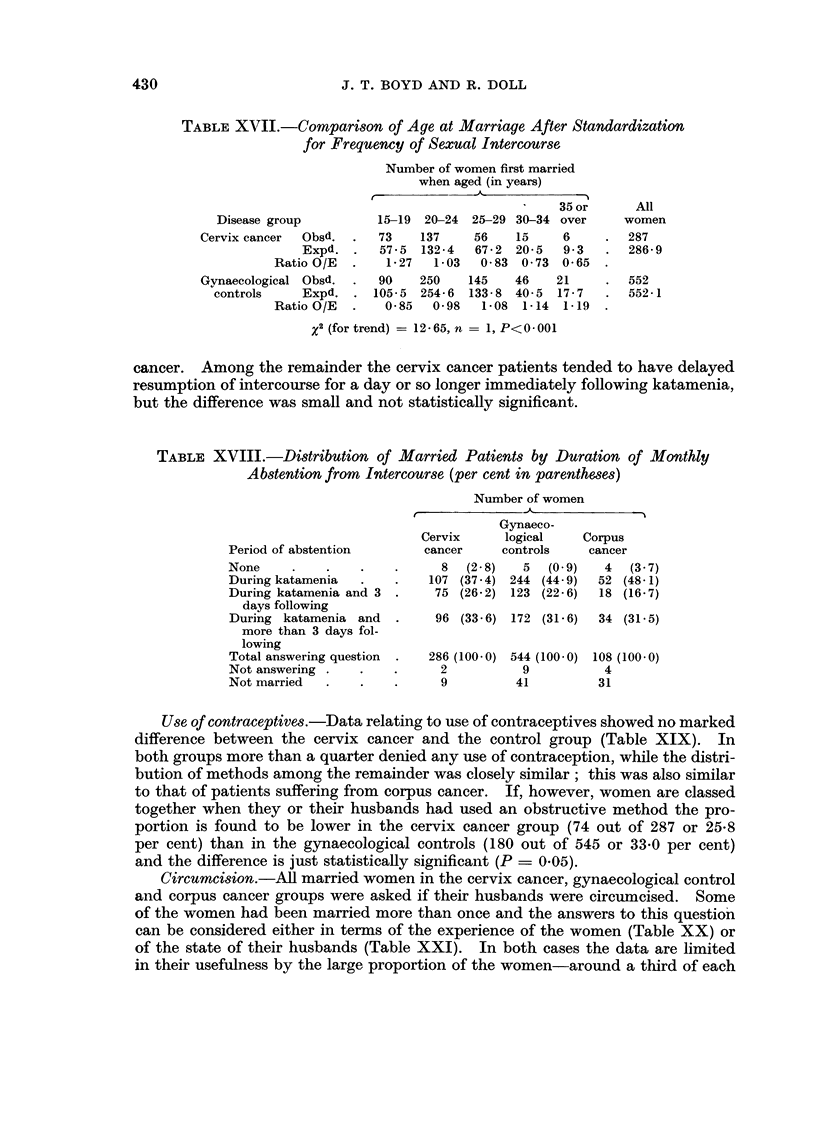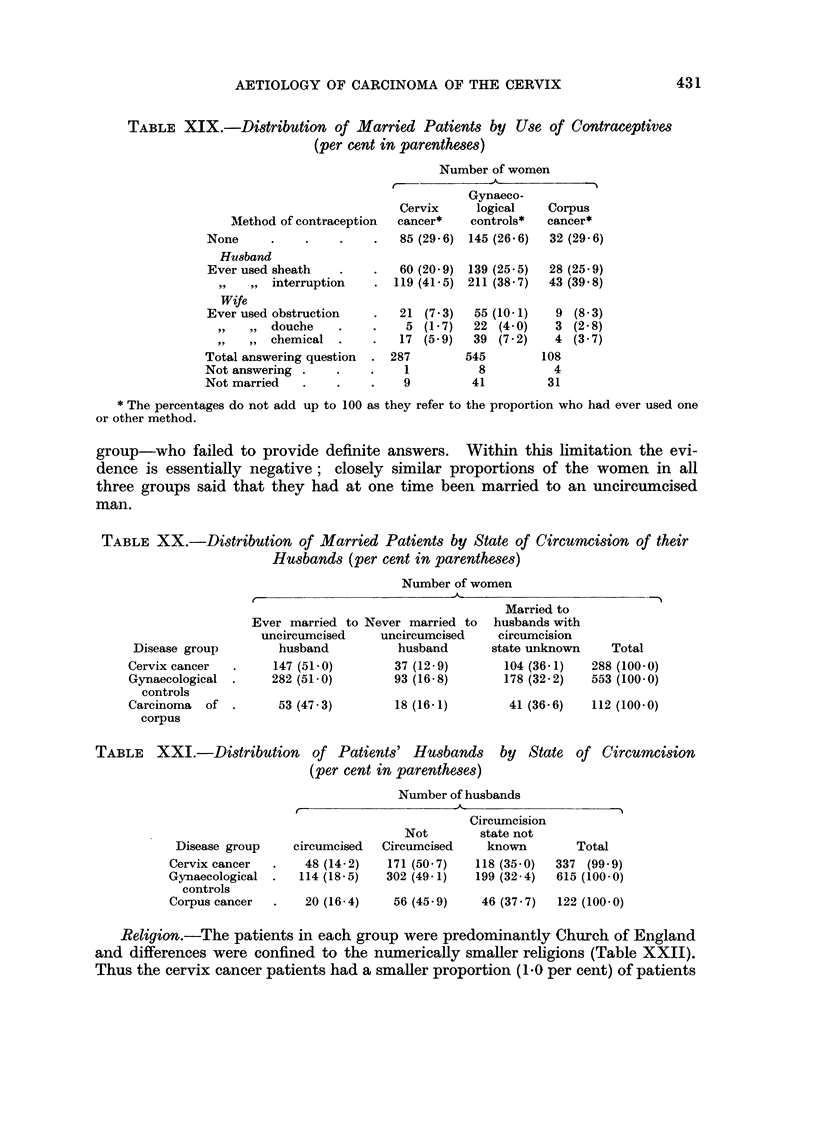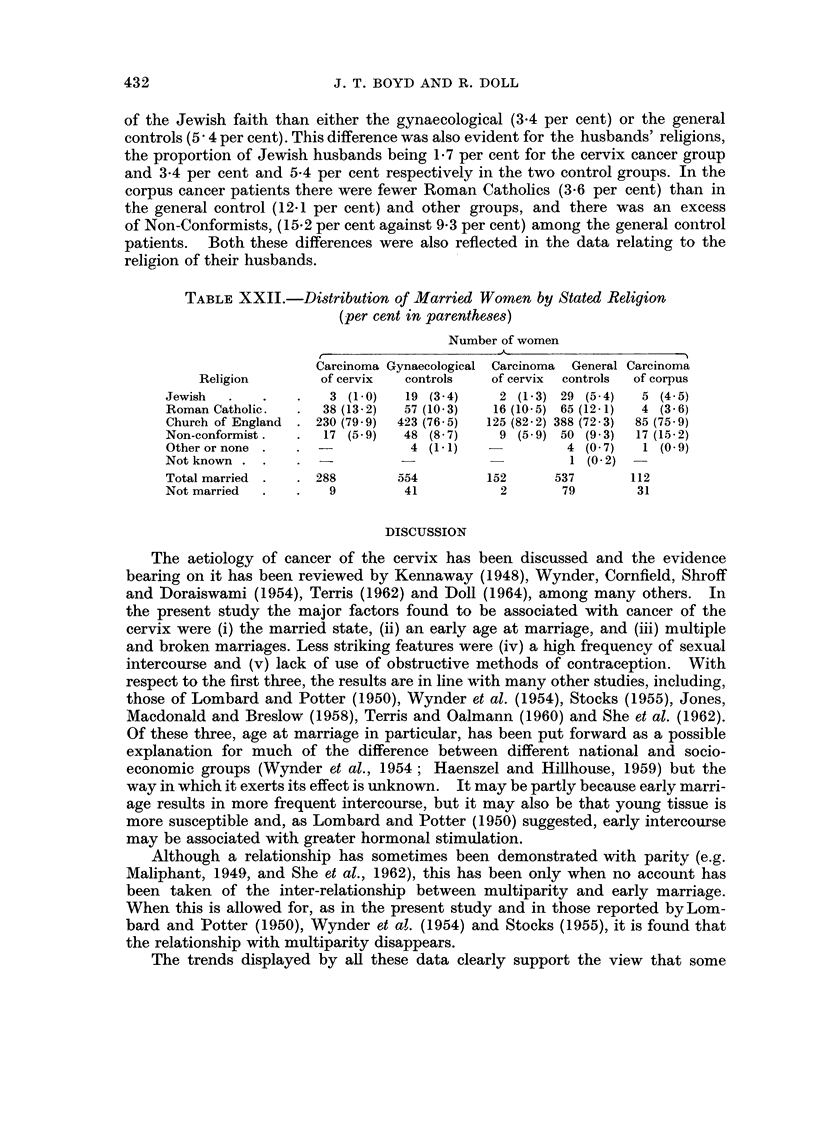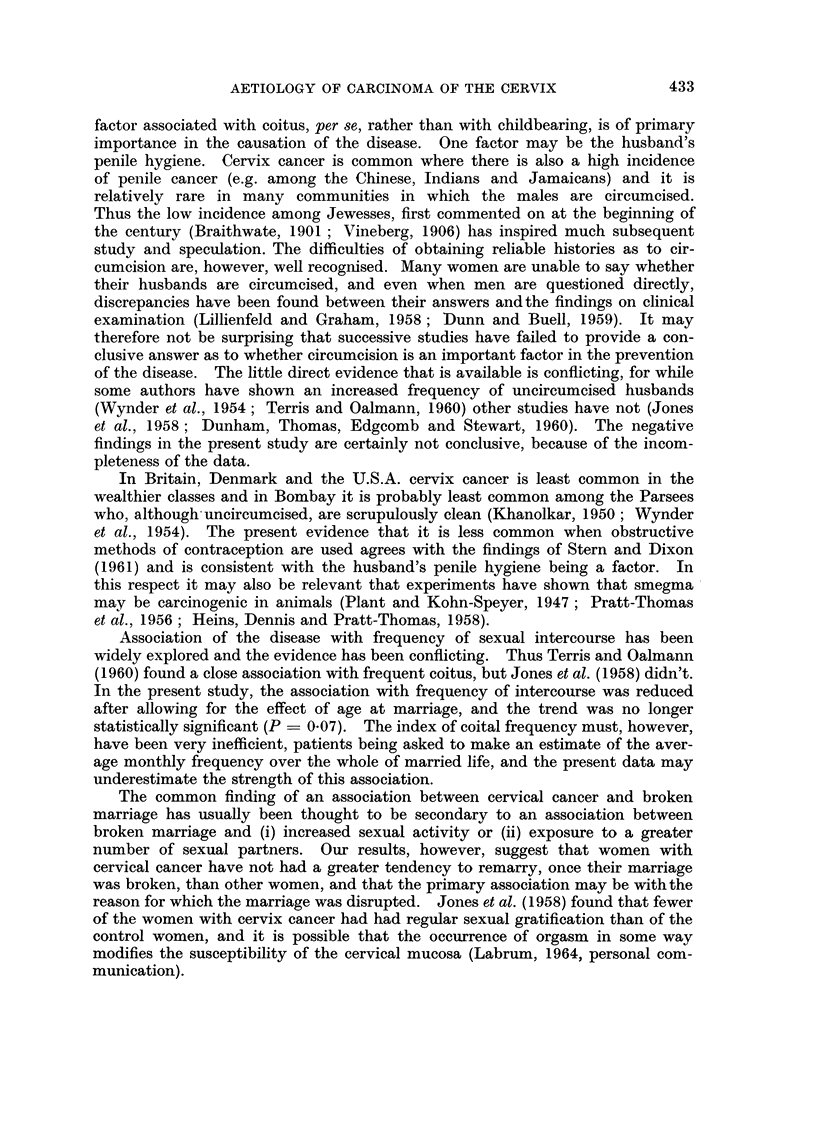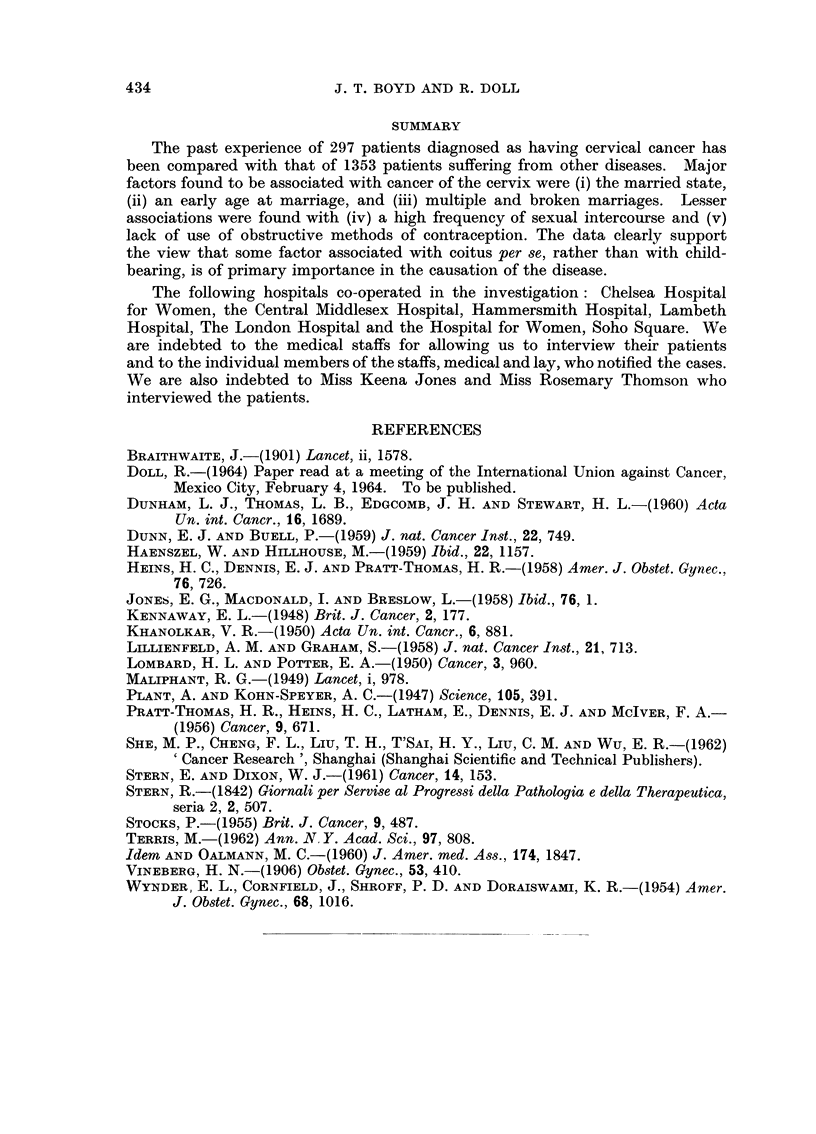# A Study of the Aetiology of Carcinoma of the Cervix Uteri

**DOI:** 10.1038/bjc.1964.49

**Published:** 1964-09

**Authors:** J. T. Boyd, R. Doll


### BRITISH JOURNAL OF CANCER

VOL. XVIII SEPTEMBER, 1964 NO. 3

### A STUDY OF THE AETIOLOGY OF CARCINOMA OF THE CERVIX UTERI

J. T. BOYD AND R. DOLL

*From the Medical Research Council's Statistical Research Unit, University College Hospital Medical School,* 115, *Gower Street, London, W.C.*1

Received for publication May 4, 1964

As a cause of death cancer of the cervix uteri is one of the less important cancers in Britain. In 1962 it was responsible for 5 per cent of all the deaths due to cancer in women, which is less than the proportion attributed to cancer of the breast (20 per cent), stomach (13 per cent), colon (12 per cent), bronchus (7 per cent) or ovary (6 per cent). It has, however, the distinction of being prevalent throughout the world and is common in many countries in which cancer is otherwise relatively rare. Only in Israel is it thought that the mortality is substantially lower than in Britain.*

Partly because of its widespread prevalence and the ease with which it can be diagnosed and partly, perhaps, because of its apparent relationship to marriage and childbearing, cancer of the cervix has been the subject of a great many aetiological studies. As long ago as 1842, Stern examined the records of cancer deaths in Verona and concluded that cancer of the uterus (which in his case would have been mostly cancer of the cervix) bore an inverse relationship to cancer of the breast and that the married state increase the number of uterine cancers.

In more recent years attention has been attracted by the rarity of the disease among Jewesses (Kennaway, 1948) and by the relative rarity among Moslems in India in comparison with its prevalence among Hindus. As a result it has been suggested that the disease could be largely prevented if all men were circumcised. It has been noted also that in several countries the disease is more prevalent among the poorer women than among the wealthier.

The present study, which was carried out between 1951 and 1953, was begun at the instigation of Sir Ernest Kennaway in the hope of elucidating some of these relationships.

### MATERIAL AND METHOD

Histories obtained from patients suffering from carcinoma of the cervix have been compared with those obtained from other patients suffering from other diseases. The patients were all under treatment at one or other of six hospitals, four of which were general hospitals (group A) while two specialised in

* Reliable mortality data for cancer of the cervix are not widely available, as cancer of the cervix and cancer of the corpus uteri are not always distinguished on death certificates.

gynaecology and obstetrics (group B).* Patients diagnosed as having cancer of the female genital tract were notified to the Statistical Research Unit, when they were visited and interviewed by one of two full time research almoners, the results of the interview being recorded at the time on a standard form.

To obtain control data, each patient with cervix cancer was matched for age (within the same 5 year age group) with *two* other patients under treatment in the gynaecological wards of the same hospitals, excluding only patients with cancer of other parts of the genital tract. These patients are referred to subsequently as gynaecological controls ; they are shown in Table I, divided according to the diagnosis on discharge from hospital. Clearly, some of the conditions were related to pregnancy while others were related to infertility and it cannot be assumed that these women were necessarily representative of the general population in respect of either their marriage or obstetric histories. A second control series was, therefore, obtained by selecting *four* other women under treatment for a non-gynaecological condition in the medical or surgical wards of the general hospitals (group A) and matched for age (within the same 5 year age group) with the cervix cancer patients in the same hospital. These " general controls " are less likely to be biased in respect of their marriage histories than the gynaecological controls and they have been used in examining the results to help interpret the differences between the cervix cancer patients and the gynaecological controls. Where similar differences are observed between cervix cancer patients and both control series it is reasonable to assume, as a working hypothesis, that the abnormality is a characteristic of the patients with cervix cancer. Data were also obtained from a fourth group of patients, not matched for age, who were under treatment for carcinoma of the corpus uteri. Histories were obtained and recorded in the same way from all patients, save that some of the more intimate questions relating to marriage were omitted when interviewing patients in the general control group.

TABLE I.—*Gynaecological Controls : Diagnoses and Numbers of Patients*

| Diagnosis | Number of patients |
|---|---|
| Prolapse, cystocoele, etc. | 292 |
| Fibroid | 62 |
| Disorder of menstrual bleeding | 48 |
| Polyp | 23 |
| Ovarian tumour (benign) | 18 |
| Cervicitis, erosion | 17 |
| Post menopausal bleeding | 17 |
| Complications of early pregnancy | 10 |
| Vaginitis and vulvitis | 9 |
| Leukoplakia | 9 |
| Malposition of uterus | 9 |
| Urethral caruncle | 9 |
| Endometriosis | 8 |
| Pelvic sepsis | 8 |
| Non-gynaecological disorder | 11 |
| Other | 44 |
| All diagnoses | 594 |

* The co-operating hospitals were The Central Middlesex Hospital, Hammersmith Hospital, The London Hospital, Lambeth Hospital (Group A) and Chelsea Hospital for Women and The Hospital for Women, Soho Square (Group B).

Altogether data were obtained from 1650 patients, of whom 297 had carcinoma of the cervix, 594 were gynaecological controls, 616 were general controls, and 143 had carcinoma of the corpus. The age distribution of the patients in each diagnostic group is shown in Table II. In this and subsequent tables comparisons have been made between :

(i) all the cervix cancer patients and all the gynaecological controls (hospitals A and B), and

(ii) the 154 cervix cancer patients seen in the general hospitals and the 616 general controls (hospitals A).

TABLE II.—*Distribution of Patients by Age*

| Age (years) | Hospitals A and B: No. of cervix cancer patients | Hospitals A and B: No. of gynaecological controls | Hospitals A: No. of cervix cancer patients | Hospitals A: No. of general controls | Hospitals A and B: No. of corpus cancer patients |
|---|---|---|---|---|---|
| <25 | 1 | 2 | — | — | — |
| 25– | 2 | 4 | — | — | 1 |
| 30– | 8 | 16 | 4 | 16 | 1 |
| 35– | 26 | 52 | 17 | 68 | 1 |
| 40– | 34 | 68 | 19 | 76 | 9 |
| 45– | 39 | 78 | 24 | 96 | 16 |
| 50– | 58 | 116 | 26 | 104 | 26 |
| 55– | 58 | 116 | 29 | 116 | 45 |
| 60–64 | 71 | 142 | 35 | 140 | 44 |
| All ages | 297 | 594 | 154 | 616 | 143 |
| Average age (years) | 51·9 | 51·9 | 51·5 | 51·5 | 55·5 |

The remaining patients with carcinoma of the body of the uterus, who were not specifically matched by age and hospital with the cervix patients, have not been used for any rigorous comparison as in (i) and (ii) above. The data for them have, however, been analysed in parallel with those for the other groups, and where appropriate, they are presented separately in the tables.

### RESULTS

*Social class.*—Occupational histories were used to allocate the patients to one or other of the Register General's five social class groups, single persons being classified according to their own occupation and married women according to the occupation of their husbands (Table III). The data suggest that hospitals in group A had rather more patients in Classes IV and V, than the hospitals in group B, but the design of the survey, using matched controls from the same hospitals, has ensured that such differences would not have any important effect on the main comparisons under study. Thus the social class distributions of the cervix cancer patients and their matched gynaecological controls (from hospitals A and B) were similar, as were those of the smaller cervix cancer group and their general controls (from hospital A). The patients with corpus cancer reflected the social class pattern of the other two groups drawn from the same hospitals (A and B).

*Menses.*—There was little difference between the cervix cancer, control and corpus cancer groups with respect to their menstrual histories (Table IV). In each group some 7 per cent of patients provided a history of irregular menstruation

TABLE III.—*Distribution of Patients by Social Class*

| Social class | Hospitals A and B: Cervix cancer patients No. | Per cent | Hospitals A and B: Gynaecological controls No. | Per cent | Hospitals A: Cervix cancer patients No. | Per cent | Hospitals A: General controls No. | Per cent | Hospitals A and B: Corpus cancer No. | Per cent |
|---|---|---|---|---|---|---|---|---|---|---|
| I | 13 | 4·4 | 25 | 4·2 | 5 | 3·2 | 23 | 3·7 | 5 | 3·5 |
| II | 33 | 11·1 | 83 | 14·0 | 16 | 10·4 | 71 | 11·5 | 18 | 12·6 |
| III | 194 | 65·3 | 377 | 63·5 | 94 | 61·0 | 353 | 57·3 | 92 | 64.3 |
| IV | 30 | 10·1 | 55 | 9·3 | 18 | 11·7 | 74 | 12·0 | 16 | 11·2 |
| V | 27 | 9·1 | 50 | 8·4 | 21 | 13·6 | 93 | 15·1 | 11 | 7·8 |
| Not stated | — | — | 4 | 0·7 | — | — | 2 | 0·3 | 1 | 0·7 |
| All classes | 297 | | 594 | | 154 | | 616 | | 143 | |

and in each the average age at menarche was close to 14 years. In comparing the distributions around these and other averages, use has been made of the design of the investigation, whereby the overall ratio of matched controls to cervix cases was 2 : 1 for the gynaecological controls and 4 : 1 for the general controls. Thus in the present comparison, the similarity of the cervix and gynaecological control groups is emphasised by the lack of any notable deviation from the ratio of 2 : 1 for each of the age at menarche sub-groups. Comparing the general controls with cervix cancer patients, breakdown by age does not show quite the same stability of the ratio. With ages 9–10 years at menarche (where the numbers are very small) and 11–12 years there are relatively more patients in the cervix cancer group than with other ages ; the difference between the groups as a whole is however, not statistically significant.

*Frequency of marriage.*—The marriage experience* of the cervix cancer patients differed from that of the patients in both the control groups (Table V). In

TABLE IV.—*Distribution of Patients by Age at Menarche*

| Age (years) | Number of women: Hospitals A and B: Cervix cancer (c) | Gynaecological controls (d) | Ratio d : c | Hospitals A: Cervix cancer (e) | General controls (f) | Ratio f : e | Hospitals A and B corpus cancer |
|---|---|---|---|---|---|---|---|
| 9–10 | 7 | 13 | 1·9 | 5 | 4 | 0·8 | 1 |
| 11–12 | 58 | 132 | 2·3 | 37 | 113 | 3·1 | 31 |
| 13–14 | 143 | 270 | 1·9 | 69 | 293 | 4·2 | 60 |
| 15–16 | 70 | 136 | 1·9 | 32 | 156 | 4·9 | 40 |
| 17 or over | 19 | 43 | 2·3 | 11 | 49 | 4·5 | 11 |
| Not stated | — | — | — | — | 1 | — | — |
| All ages | 297 | 594 | 2·0 | 154 | 616 | 4·0 | 143 |
| Average age at onset | 13·9 yrs. | 13·7 yrs. | | 13·7 yrs. | 14·0 yrs. | | 14·0 yrs. |
| Difference ±S.E. | 0·13±0·13 | | | 0·21±0·17 | | | |
| Per cent with irregular menses | 7·1 | 6·7 | | 6·5 | 7·6 | | 7·0 |
| Differences ±S.E. | 0·34±1·80 | | | 1·14±2·36 | | | |

* Throughout the present study marriage has been taken to include *de facto* marriage as well as *de jure*.

particular the cervix cancer groups contained *fewer* unmarried women (3·0 per cent and 1·3 per cent) than the control series (6·9 per cent and 12·8 per cent respectively) and in each case the difference between the cervix cancer patients and their matched controls was greater than would be expected by chance alone. It should be noted also that 3 of the 9 single women among the cervix patients reported a previous pregnancy, the comparable proportions in the other groups being 1 out of 41 (gynaecological controls) 5 out of 79 (general controls) and 0 out of 31 (corpus cancer) Again, in both main comparisons, the relative frequency of multiple marriages among patients with cervix cancer was demonstrated by the decreasing control/cervix ratio with increase in number of marriages. In contrast to the cervix cancer patients (3·0 per cent) those suffering from corpus cancer included a much higher proportion of unmarried women (21·7 per cent),

TABLE V.—*Distribution of Patients by Numbers of Times Married*

| | Number of women | | | | | | |
|---|---|---|---|---|---|---|---|
| | Hospitals A and B | | | Hospitals A | | | |
| Number of marriages | Cervix cancer (c) | Gynaecological controls (d) | Ratio d : c | Cervix cancer (e) | General controls (f) | Ratio f : e | Hospitals A and B corpus cancer |
| 0 | 9 | 41 | 4·6 | 2 | 79 | 39·5 | 31 |
| 1 | 240 | 494 | 2·1 | 131 | 482 | 3·7 | 102 |
| 2 or more | 48 | 59 | 1·2 | 21 | 55 | 2·6 | 10 |
| All women | 297 | 594 | 2·0 | 154 | 616 | 4·0 | 143 |
| Per cent not married | 3·0 | 6·9 | | 1·3 | 12·8 | | 21·7 |
| Difference ±S.E. | 3·87† ±1·64 | | | 11·52* ±2·75 | | | |

† $P<0·05$.
* $P<0·01$.

*Age at marriage*—Differences in marriage histories were again apparent in the data relating to age at marriage (Table VI) Thus the average age at marriage of the 288 married women with carcinoma of the cervix was 22·7 years compared with 23·9 years for the 553 married women in the gynaecological control group. A similar difference was evident in the comparison between cervix cancer patients and the general controls. In both comparisons the difference with respect to average age at marriage was statistically significant (in each case $P<0·01$). Married patients with corpus cancer, on the other hand, had on average, married at a later age than patients in the other groups.

*Broken marriage.*—The frequency of broken first marriage—whether due to the death of the husband or to separation or divorce—is shown in Table VII. For women married under 20 years of age the frequency was high and there was no striking difference between the groups. With later marriage, however, the proportion broken was substantially higher for the cervix cancer patients than for the controls and the difference between them was so great that the total proportions of broken marriages (standardized for age) were significantly different ($P<0·001$ for cervix cancer and gynaecological controls ; $P<0·05$ for cervix cancer and general controls). Clearly, therefore, the high proportion of broken marriages in the cervix cancer group (45 per cent) cannot be explained wholly on the grounds of early marriage.

TABLE VI.—*Distribution of Patients by Age at Marriage*

| | Number of married women | | | | | | |
|---|---|---|---|---|---|---|---|
| | Hospitals A and B | | | Hospitals A | | | |
| Age (years) | Cervix cancer (c) | Gynaecological controls (d) | Ratio d : c | Cervix cancer (e) | General controls (f) | Ratio f : e | Hospitals A and B corpus cancer |
| 15– | 73 | 91 | 1·2 | 38 | 107 | 2·1 | 15 |
| 20– | 138 | 250 | 1·8 | 80 | 249 | 3·1 | 36 |
| 25– | 56 | 145 | 2·6 | 21 | 121 | 5·8 | 44 |
| 30– | 15 | 46 | 3·1 | 9 | 35 | 3·9 | 10 |
| 35 or over | 6 | 21 | 3·5 | 4 | 25 | 6·3 | 7 |
| All ages | 288 | 553 | 1·9 | 152 | 537 | 3·5 | 112 |
| Average age at marriage (years) | 22·7 | 23·9 | | 22·5 | 23·8 | | 25·3 |
| Difference ±S.E. | 1·26*±0·36 | | | 1·26*±0·49 | | | |

* $P<0\cdot01$.

*Remarriage.*—Table VIII shows that remarriage after the first marriage had been broken was practically the same in all diagnostic groups. The high proportion of multiple marriages among the cervix cancer patients shown in Table V should, therefore, be attributed to a greater frequency of broken marriages and not to a particular desire for remarriage after the first marriage had been broken.

*Duration of marriage.*—Since the cervix cancer and the control patients were matched for age at the time of interview and the cervix cancer patients had, on average, married at an earlier age, it might have been supposed that the duration of marriage for the cervix cancer patients would have been longer. In fact, the differences were very slight, and the longest average duration of marriage was actually recorded for the gynaecological controls. The explanation lies in the fact that marriage in the cervix cancer group was more often broken. When broken and unbroken marriages are considered separately the average duration of marriage is found to be slightly longer in each case in the cervix cancer group (Table IX) ; in no case, however, is the difference statistically significant.

TABLE IX.—*Duration of Marriage*

| | Average duration of marriage (years) | | | |
|---|---|---|---|---|
| | Hospitals A and B | | Hospitals A | |
| Marriage | Cervix cancer patients | Gynaecological controls | Cervix cancer patients | General controls |
| Broken | 22·1 | 21·6 | 20·9 | 20·7 |
| Unbroken | 26·5 | 25·8 | 26·8 | 25·4 |
| All marriages | 24·5 | 24·6 | 24·4 | 23·9 |

*Age at pregnancy.*—In view of the findings with respect to age at marriage it is not surprising that the cervix and control groups also differ in age at first pregnancy (Table X). A higher proportion of cervix cancer patients gave a history of pregnancy at a young age and, conversely, the proportion of women having their first pregnancy at ages 30 years and over was considerably higher in the control groups. Direct comparison of average ages at first pregnancy

TABLE VII.—*Proportion of Broken First Marriages, by Age at Marriage*

| Age at first marriage (years) | Hospitals A and B | | | | | | Hospitals A | | | | | |
|---|---|---|---|---|---|---|---|---|---|---|---|---|
| | Cervix cancer | | | Gynaecological controls | | | Cervix cancer | | | General controls | | |
| | Married women No. | Broken marriages No. | Broken marriages Per cent | Married women No. | Broken marriages No. | Broken marriages Per cent | Married women No. | Broken marriages No. | Broken marriages Per cent | Married women No. | No. | Per cent |
| 15– | 73 | 35 | 47·9 | 91 | 47 | 51·6 | 38 | 15 | 39·5 | 107 | 49 | 45·8 |
| 20– | 138 | 67 | 48·6 | 250 | 79 | 31·6 | 80 | 36 | 45·0 | 249 | 69 | 27·7 |
| 25– | 56 | 18 | 32·1 | 145 | 29 | 20·0 | 21 | 6 | 28·6 | 121 | 33 | 27·3 |
| 30 or over | 21 | 9 | 42·9 | 67 | 7 | 10·4 | 13 | 6 | 46·2 | 60 | 13 | 21·7 |
| All ages | 288 | 129 | 44·8 | 553 | 162 | 29·3 | 152 | 63 | 41·4 | 537 | 164 | 30·5 |

TABLE VIII.—*Proportion of Remarriages, by Age at First Marriage*

| Age at first marriage (years) | Hospitals A and B | | | | | | Hospitals A | | | | | |
|---|---|---|---|---|---|---|---|---|---|---|---|---|
| | Cervix cancer | | | Gynaecological controls | | | Cervix cancer | | | General controls | | |
| | Broken marriage No. | Remarriage No. | Remarriage Per cent | Broken marriage No. | Remarriage No. | Remarriage Per cent | Broken marriage No. | Remarriage No. | Remarriage Per cent | Broken marriage No. | No. | Per cent |
| 15– | 35 | 17 | 48·6 | 47 | 23 | 48·9 | 15 | 8 | 53·3 | 49 | 29 | 59·2 |
| 20– | 67 | 24 | 35·8 | 79 | 29 | 36·7 | 36 | 10 | 27·8 | 69 | 22 | 31·9 |
| 25 or over | 27 | 7 | 25·9 | 36 | 7 | 19·4 | 12 | 3 | 25·0 | 46 | 4 | 8·7 |
| All ages | 129 | 48 | 37·2 | 162 | 59 | 36·4 | 63 | 21 | 33·3 | 164 | 55 | 33·5 |

demonstrated this to be significantly lower among the patients with cervix cancer. A notable feature of the corpus cancer patients was the high proportion that had never at any time been pregnant; that is, 45 per cent (64 out of 143) compared with 21 per cent in the general controls, 15 per cent in the gynaecological controls, and 11 per cent in the cervix cancer patients.

TABLE X.—*Distribution of Patients by age At First Pregnancy*

| | Number of women | | | | | | |
|---|---|---|---|---|---|---|---|
| | Hospitals A and B | | | Hospitals A | | | |
| Age at first pregnancy (years) | Cervix cancer (c) | Gynaecological controls (d) | Ratio d : c | Cervix cancer (e) | General controls (f) | Ratio f : e | Hospitals A and B corpus cancer |
| 15– | 46 | 54 | 1·2 | 22 | 59 | 2·7 | 10 |
| 20– | 133 | 207 | 1·6 | 76 | 209 | 2·8 | 22 |
| 25– | 63 | 139 | 2·2 | 29 | 131 | 4·5 | 31 |
| 30– | 18 | 79 | 4·4 | 10 | 61 | 6·1 | 10 |
| 35 or over | 3 | 23 | 7·7 | 1 | 23 | 23·0 | 6 |
| Not stated | — | — | | — | 1 | | — |
| All ages | 263 | 502 | 1·9 | 138 | 484 | 3·5 | 79 |
| No Pregnancy | 34 | 92 | | 16 | 132 | | 64 |
| Average age at first pregnancy (years) | 23·1 | 25·1 | | 23·1 | 24·7 | | 25·7 |
| Difference ±S.E. | | 2·01*±0·36 | | | 1·63*±0·46 | | |

* $P<0\cdot01$.

*Number of pregnancies.*—The numbers of children born to married women in each of the study groups is shown in Table XI. The proportions of childless married women were very similar in the cervix cancer and the two control groups. Thus 38 (13 per cent) of 288 married women with cervix cancer had had no children, 65 (12 per cent) of the 553 women in the gynaecological control group and 13 per cent and 14 per cent respectively in the cervix and general control patients from the A hospitals. On the other hand, the cervix patients contained relatively more women with larger families—21 per cent of them (60 out of 288) having had 5 or more children compared with the 13 per cent (70 out of 553) in the gynaecological controls. The differences between the average number of children for the cervix cancer patients and the two control groups were small; the difference from the gynaecological controls was clearly significant ($P<0\cdot01$) but the difference from the general control patients was not ($P = 0\cdot06$). The data relating to patients with corpus cancer once again demonstrated their relatively low parity, in that more than one third of the married women in this group had had no children and only 7 (6 per cent) had a family of 5 or more.

The apparent relationships between carcinoma of the cervix and (i) age at marriage and (ii) number of children were examined further by eliminating the effect of each of these factors in turn from comparisons involving the other. For example, the combined experience of the cervix and gynaecological control groups showed that, of 78 mothers with 5 or 6 children, 30 were married at ages 15–19 years, 36 were married at ages 20–24 years and 12 at ages 25–29 years. It might, therefore, be expected that (30/78) × 33 or 12·7 of the 33 cervix cancer patients with 5 or 6 children would have been married at ages 15–19 years; and

TABLE XI.—*Distribution of Married Patients by Number of Children*

| Number of children | Hospitals A and B: Cervix cancer (c) | Hospitals A and B: Gynaecological controls (d) | Ratio d : c | Hospitals A: Cervix cancer (e) | Hospitals A: General controls (f) | Ratio f : e | Hospitals A and B corpus cancer |
|---|---|---|---|---|---|---|---|
| 0 | 38 | 65 | 1·7 | 20 | 75 | 3·8 | 39 |
| 1–2 | 121 | 278 | 2·3 | 58 | 239 | 4·1 | 52 |
| 3–4 | 69 | 140 | 2·0 | 39 | 136 | 3·5 | 14 |
| 5–6 | 33 | 45 | 1·4 | 19 | 48 | 2·8 | 4 |
| 7–8 | 17 | 14 | 0·8 | 11 | 27 | 2·5 | 2 |
| 9 or over | 10 | 11 | 1·1 | 5 | 12 | 2·4 | 1 |
| All numbers | 288 | 553 | 1·9 | 152 | 537 | 3·5 | 112 |
| Not married | 9 | 41 | | 2 | 79 | | 31 |
| Average No. of children | 2·9 | 2·5 | | 3·1 | 2·7 | | 1·5 |
| Difference ±S.E. | 0·43*±0·16 | | | 0·41±0·22 | | | |

(Number of women)

* $P<0 \cdot 01$.

(36/78) × 33 or 15·2 would have been married at ages 25–29 years. In a like manner the number of women " expected " to have been married at different ages was calculated for each of the other parity groups. By adding the experience of all the parity groups an estimate was obtained of the " expected " distribution of ages at marriage in which allowance had been made for the parity of the cervix cancer patients and a similar calculation provided the " expected " distribution of ages at marriage for the gynaecological controls.

On comparison of the two groups (Table XII) the excess of younger marriages among cervix cancer patients remained evident (211 patients married at ages under 25 years compared with 193 " expected "), and the observed/expected ratio displayed a consistent downward trend with increasing age at marriage.

TABLE XII.—*Comparison of Ages at Marriage After Standardization for Number of Children*

| Disease group | | Number of women first married when aged (in years): 15–19 | 20–24 | 25–29 | 30–34 | 35 or over | All women |
|---|---|---|---|---|---|---|---|
| Cervix cancer | Obsd. | 73 | 138 | 56 | 15 | 6 | 288 |
| | Expd. | 61·2 | 131·8 | 65·7 | 19·9 | 9·3 | 287·9 |
| | Ratio O/E | 1·19 | 1·05 | 0·85 | 0·75 | 0·65 | |
| Gynaecological controls | Obsd. | 91 | 251 | 144 | 46 | 21 | 553 |
| | Expd. | 102·8 | 257·2 | 134·3 | 41·1 | 17·7 | 553·1 |
| | Ratio O/E | 0·89 | 0·98 | 1·07 | 1·12 | 1·19 | |

$\chi^2$ (for trend) $= 9 \cdot 57$, $n = 1$, $P<0 \cdot 005$

It was clear therefore that, after allowing for possible parity effects, the differences between the groups in respect of age at marriage remained significant. A comparison between the groups was then made with respect to the number of children after standardisation for age at marriage (Table XIII). There were still some differences between the numbers of children " observed " and " expected ", but they were less regular and there was no statistically significant trend in the ratio between the groups. Thus, in contrast to the findings for age at marriage, the analysis suggests the differences that had been noted with respect to parity were a result of the differences in age at marriage and they provide no evidence of an independent effect of parity. Analysis of the data for all pregnancies rather than for children provides essentially similar results (Doll, 1964).

TABLE XIII.—*Comparison of Number of Children After Standardization for Ages at Marriage*

| Disease group | | Number of women with different numbers of children: 0 | 1–2 | 3–4 | 5–6 | 7–8 | 9 or over | All women |
|---|---|---|---|---|---|---|---|---|
| Cervix cancer | Obsd. | 38 | 121 | 69 | 33 | 17 | 10 | 288 |
| | Expd. | 30·7 | 132·6 | 74·3 | 29·6 | 12·1 | 8·7 | 288·0 |
| | Ratio O/E | 1·24 | 0·92 | 0·93 | 1·11 | 1·40 | 1·15 | |
| Gynaecological controls | Obsd. | 65 | 278 | 140 | 45 | 14 | 11 | 553 |
| | Expd. | 72·3 | 266·4 | 134·7 | 48·4 | 18·9 | 12·3 | 553·0 |
| | Ratio O/E | 0·90 | 1·04 | 1·04 | 0·93 | 0·74 | 0·89 | |

$\chi^2$ (for trend) $= 0\cdot66$, $n = 1$, $P > 0\cdot1$

*Frequency of sexual intercourse.*—An index of sexual activity was obtained by asking each married patient to express her own experience in terms of an average monthly frequency of intercourse throughout married life. Analysis of these data suggests that cervix cancer patients experience more frequent intercourse than women in the control group (Table XIV). Thus 99, or just over one third, of the cervix cancer patients reported frequencies of 8 (or more) per month against 137, or just under one quarter, of the gynaecological control patients. Examination of the average frequencies (6·2 and 5·2 per month respectively) showed the same trend and the difference between the means was statistically significant ($P < 0{\cdot}01$). The data for the control group show, however, that there was a negative association between frequency of intercourse and age at marriage, demonstrated by the trend in the average frequency from 6·5 per month for those married at ages under 20 years, to 5·3 per month for women married between 20 and 24 years and 4·5 per month for those married at 25 and over (Table XV). It is therefore necessary to standardize for age at marriage, before comparing the groups with respect to frequency of intercourse. After standardization the results (Table XIV) show that the difference between the two groups is less marked ; the trend in the ratio of cervix cancer to control patients with increasing frequency of intercourse still persists, but it is no longer statistically significant ($P = 0{\cdot}07$). In contrast, the excess of younger marriages is hardly affected by standardizing for frequency of intercourse and the relationship remains close (Table XVII).

*Intercourse in relation to katamenia.*—Questions were also asked about the occurrence of intercourse in relation to katamenia (Table XVIII). The proportion of patients denying any restriction of intercourse was small (around 2 per cent) and in this respect the experience of the cervix cancer patients lay intermediate between that of the gynaecological control group and the patients with corpus

TABLE XIV.—*Distribution of Married Patients by Frequency of Sexual Intercourse*

| Frequency per month | Number of women: Cervix cancer (c) | Gynaecological controls (d) | Ratio d : c | Corpus cancer |
|---|---|---|---|---|
| 0–3 | 97 | 195 | 2·0 | 41 |
| 4–7 | 91 | 220 | 2·4 | 43 |
| 8–11 | 68 | 100 | 1·5 | 19 |
| 12–15 | 16 | 18 | 1·1 | 7 |
| 16 or over | 15 | 19 | 1·3 | 1 |
| Not stated | 1 | 1 | — | 1 |
| All frequencies | 288 | 553 | 1·9 | 112 |
| Not married | 9 | 41 | — | 31 |
| Average frequency per month | 6·2 | 5·2 | | 5·0 |
| Difference | 0·98*±0·37 | | | |

* $P<0.01$

TABLE XV.—*Distribution of Married Patients in Gynaecological Control Group by Frequency of Sexual Intercourse and Age at Marriage (per cent in parentheses)*

| Average frequency per month | Number of women first married when aged (in years): 15–19 | | 20–24 | | 25 or over | |
|---|---|---|---|---|---|---|
| 0–3 | 28 | (31·1) | 84 | (33·6) | 83 | (39·2) |
| 4–7 | 30 | (57·8) | 103 | (59·6) | 87 | (56·1) |
| 8–11 | 22 | | 46 | | 32 | |
| 12–15 | 5 | (5·6) | 9 | (3·6) | 4 | (1·9) |
| 16 or over | 5 | (5·6) | 8 | (3·2) | 6 | (2·8) |
| All frequencies | 90 | (100·1) | 250 | (100·0) | 212 | (100·0) |
| Not stated | 1 | | 0 | | 0 | |
| Average frequency | 6·5 | | 5·3 | | 4·5 | |

TABLE XVI.—*Comparison of Frequency of Sexual Intercourse After Standardization for Age at Marriage*

| Disease group | | Number of women having different months frequencies of sexual intercourse: 0–3 | 4–7 | 8–11 | 12–15 | 16 or over | All women |
|---|---|---|---|---|---|---|---|
| Cervix cancer | Obsd. | 97 | 91 | 68 | 16 | 15 | 287 |
| | Expd. | 98·1 | 105·1 | 59·6 | 11·8 | 12·3 | 286·9 |
| | Ratio O/E | 0·99 | 0·87 | 1·14 | 1·36 | 1·22 | |
| Gynaecological controls | Obsd. | 195 | 220 | 100 | 18 | 19 | 552 |
| | Expd. | 193·9 | 205·9 | 108·4 | 22·2 | 21·7 | 552·1 |
| | Ratio O/E | 1·01 | 1·07 | 0·92 | 0·81 | 0·88 | |

$\chi^2$ (for trend) $= 3.27, n = 1, P = 0.07$

TABLE XVII.—*Comparison of Age at Marriage After Standardization for Frequency of Sexual Intercourse*

| Disease group | | Number of women first married when aged (in years) 15–19 | 20–24 | 25–29 | 30–34 | 35 or over | All women |
|---|---|---|---|---|---|---|---|
| Cervix cancer | Obsd. | 73 | 137 | 56 | 15 | 6 | 287 |
| | Expd. | 57·5 | 132·4 | 67·2 | 20·5 | 9·3 | 286·9 |
| | Ratio O/E | 1·27 | 1·03 | 0·83 | 0·73 | 0·65 | |
| Gynaecological controls | Obsd. | 90 | 250 | 145 | 46 | 21 | 552 |
| | Expd. | 105·5 | 254·6 | 133·8 | 40·5 | 17·7 | 552·1 |
| | Ratio O/E | 0·85 | 0·98 | 1·08 | 1·14 | 1·19 | |

$\chi^2$ (for trend) $= 12 \cdot 65$, $n = 1$, $P < 0 \cdot 001$

cancer. Among the remainder the cervix cancer patients tended to have delayed resumption of intercourse for a day or so longer immediately following katamenia, but the difference was small and not statistically significant.

TABLE XVIII.—*Distribution of Married Patients by Duration of Monthly Abstention from Intercourse (per cent in parentheses)*

| Period of abstention | Number of women: Cervix cancer | Gynaecological controls | Corpus cancer |
|---|---|---|---|
| None | 8 (2·8) | 5 (0·9) | 4 (3·7) |
| During katamenia | 107 (37·4) | 244 (44·9) | 52 (48·1) |
| During katamenia and 3 days following | 75 (26·2) | 123 (22·6) | 18 (16·7) |
| During katamenia and more than 3 days following | 96 (33·6) | 172 (31·6) | 34 (31·5) |
| Total answering question | 286 (100·0) | 544 (100·0) | 108 (100·0) |
| Not answering | 2 | 9 | 4 |
| Not married | 9 | 41 | 31 |

*Use of contraceptives.*—Data relating to use of contraceptives showed no marked difference between the cervix cancer and the control group (Table XIX). In both groups more than a quarter denied any use of contraception, while the distribution of methods among the remainder was closely similar ; this was also similar to that of patients suffering from corpus cancer. If, however, women are classed together when they or their husbands had used an obstructive method the proportion is found to be lower in the cervix cancer group (74 out of 287 or 25·8 per cent) than in the gynaecological controls (180 out of 545 or 33·0 per cent) and the difference is just statistically significant ($P = 0{\cdot}05$).

*Circumcision.*—All married women in the cervix cancer, gynaecological control and corpus cancer groups were asked if their husbands were circumcised. Some of the women had been married more than once and the answers to this question can be considered either in terms of the experience of the women (Table XX) or of the state of their husbands (Table XXI). In both cases the data are limited in their usefulness by the large proportion of the women—around a third of each

TABLE XIX.—*Distribution of Married Patients by Use of Contraceptives (per cent in parentheses)*

| Method of contraception | Number of women: Cervix cancer* | Gynaecological controls* | Corpus cancer* |
|---|---|---|---|
| None | 85 (29·6) | 145 (26·6) | 32 (29·6) |
| *Husband* | | | |
| Ever used sheath | 60 (20·9) | 139 (25·5) | 28 (25·9) |
| ,, ,, interruption | 119 (41·5) | 211 (38·7) | 43 (39·8) |
| *Wife* | | | |
| Ever used obstruction | 21 (7·3) | 55 (10·1) | 9 (8·3) |
| ,, ,, douche | 5 (1·7) | 22 (4·0) | 3 (2·8) |
| ,, ,, chemical | 17 (5·9) | 39 (7·2) | 4 (3·7) |
| Total answering question | 287 | 545 | 108 |
| Not answering | 1 | 8 | 4 |
| Not married | 9 | 41 | 31 |

* The percentages do not add up to 100 as they refer to the proportion who had ever used one or other method.

group—who failed to provide definite answers. Within this limitation the evidence is essentially negative; closely similar proportions of the women in all three groups said that they had at one time been married to an uncircumcised man.

TABLE XX.—*Distribution of Married Patients by State of Circumcision of their Husbands (per cent in parentheses)*

| Disease group | Number of women: Ever married to uncircumcised husband | Never married to uncircumcised husband | Married to husbands with circumcision state unknown | Total |
|---|---|---|---|---|
| Cervix cancer | 147 (51·0) | 37 (12·9) | 104 (36·1) | 288 (100·0) |
| Gynaecological controls | 282 (51·0) | 93 (16·8) | 178 (32·2) | 553 (100·0) |
| Carcinoma of corpus | 53 (47·3) | 18 (16·1) | 41 (36·6) | 112 (100·0) |

TABLE XXI.—*Distribution of Patients' Husbands by State of Circumcision (per cent in parentheses)*

| Disease group | Number of husbands: circumcised | Not Circumcised | Circumcision state not known | Total |
|---|---|---|---|---|
| Cervix cancer | 48 (14·2) | 171 (50·7) | 118 (35·0) | 337 (99·9) |
| Gynaecological controls | 114 (18·5) | 302 (49·1) | 199 (32·4) | 615 (100·0) |
| Corpus cancer | 20 (16·4) | 56 (45·9) | 46 (37·7) | 122 (100·0) |

*Religion.*—The patients in each group were predominantly Church of England and differences were confined to the numerically smaller religions (Table XXII). Thus the cervix cancer patients had a smaller proportion (1·0 per cent) of patients of the Jewish faith than either the gynaecological (3·4 per cent) or the general controls (5·4 per cent). This difference was also evident for the husbands' religions, the proportion of Jewish husbands being 1·7 per cent for the cervix cancer group and 3·4 per cent and 5·4 per cent respectively in the two control groups. In the corpus cancer patients there were fewer Roman Catholics (3·6 per cent) than in the general control (12·1 per cent) and other groups, and there was an excess of Non-Conformists, (15·2 per cent against 9·3 per cent) among the general control patients. Both these differences were also reflected in the data relating to the religion of their husbands.

TABLE XXII.—*Distribution of Married Women by Stated Religion (per cent in parentheses)*

| | Number of women | | | | |
|---|---|---|---|---|---|
| Religion | Carcinoma of cervix | Gynaecological controls | Carcinoma of cervix | General controls | Carcinoma of corpus |
| Jewish | 3 (1·0) | 19 (3·4) | 2 (1·3) | 29 (5·4) | 5 (4·5) |
| Roman Catholic | 38 (13·2) | 57 (10·3) | 16 (10·5) | 65 (12·1) | 4 (3·6) |
| Church of England | 230 (79·9) | 423 (76·5) | 125 (82·2) | 388 (72·3) | 85 (75·9) |
| Non-conformist | 17 (5·9) | 48 (8·7) | 9 (5·9) | 50 (9·3) | 17 (15·2) |
| Other or none | — | 4 (1·1) | — | 4 (0·7) | 1 (0·9) |
| Not known | — | — | — | 1 (0·2) | — |
| Total married | 288 | 554 | 152 | 537 | 112 |
| Not married | 9 | 41 | 2 | 79 | 31 |

### DISCUSSION

The aetiology of cancer of the cervix has been discussed and the evidence bearing on it has been reviewed by Kennaway (1948), Wynder, Cornfield, Shroff and Doraiswami (1954), Terris (1962) and Doll (1964), among many others. In the present study the major factors found to be associated with cancer of the cervix were (i) the married state, (ii) an early age at marriage, and (iii) multiple and broken marriages. Less striking features were (iv) a high frequency of sexual intercourse and (v) lack of use of obstructive methods of contraception. With respect to the first three, the results are in line with many other studies, including, those of Lombard and Potter (1950), Wynder *et al.* (1954), Stocks (1955), Jones, Macdonald and Breslow (1958), Terris and Oalmann (1960) and She *et al.* (1962). Of these three, age at marriage in particular, has been put forward as a possible explanation for much of the difference between different national and socio-economic groups (Wynder *et al.*, 1954 ; Haenszel and Hillhouse, 1959) but the way in which it exerts its effect is unknown. It may be partly because early marriage results in more frequent intercourse, but it may also be that young tissue is more susceptible and, as Lombard and Potter (1950) suggested, early intercourse may be associated with greater hormonal stimulation.

Although a relationship has sometimes been demonstrated with parity (e.g. Maliphant, 1949, and She *et al.*, 1962), this has been only when no account has been taken of the inter-relationship between multiparity and early marriage. When this is allowed for, as in the present study and in those reported by Lombard and Potter (1950), Wynder *et al.* (1954) and Stocks (1955), it is found that the relationship with multiparity disappears.

The trends displayed by all these data clearly support the view that some factor associated with coitus, *per se*, rather than with childbearing, is of primary importance in the causation of the disease. One factor may be the husband's penile hygiene. Cervix cancer is common where there is also a high incidence of penile cancer (e.g. among the Chinese, Indians and Jamaicans) and it is relatively rare in many communities in which the males are circumcised. Thus the low incidence among Jewesses, first commented on at the beginning of the century (Braithwate, 1901 ; Vineberg, 1906) has inspired much subsequent study and speculation. The difficulties of obtaining reliable histories as to circumcision are, however, well recognised. Many women are unable to say whether their husbands are circumcised, and even when men are questioned directly, discrepancies have been found between their answers and the findings on clinical examination (Lillienfeld and Graham, 1958 ; Dunn and Buell, 1959). It may therefore not be surprising that successive studies have failed to provide a conclusive answer as to whether circumcision is an important factor in the prevention of the disease. The little direct evidence that is available is conflicting, for while some authors have shown an increased frequency of uncircumcised husbands (Wynder *et al.*, 1954 ; Terris and Oalmann, 1960) other studies have not (Jones *et al.*, 1958 ; Dunham, Thomas, Edgcomb and Stewart, 1960). The negative findings in the present study are certainly not conclusive, because of the incompleteness of the data.

In Britain, Denmark and the U.S.A. cervix cancer is least common in the wealthier classes and in Bombay it is probably least common among the Parsees who, although uncircumcised, are scrupulously clean (Khanolkar, 1950 ; Wynder *et al.*, 1954). The present evidence that it is less common when obstructive methods of contraception are used agrees with the findings of Stern and Dixon (1961) and is consistent with the husband's penile hygiene being a factor. In this respect it may also be relevant that experiments have shown that smegma may be carcinogenic in animals (Plant and Kohn-Speyer, 1947 ; Pratt-Thomas *et al.*, 1956 ; Heins, Dennis and Pratt-Thomas, 1958).

Association of the disease with frequency of sexual intercourse has been widely explored and the evidence has been conflicting. Thus Terris and Oalmann (1960) found a close association with frequent coitus, but Jones *et al.* (1958) didn't. In the present study, the association with frequency of intercourse was reduced after allowing for the effect of age at marriage, and the trend was no longer statistically significant ($P = 0{\cdot}07$). The index of coital frequency must, however, have been very inefficient, patients being asked to make an estimate of the average monthly frequency over the whole of married life, and the present data may underestimate the strength of this association.

The common finding of an association between cervical cancer and broken marriage has usually been thought to be secondary to an association between broken marriage and (i) increased sexual activity or (ii) exposure to a greater number of sexual partners. Our results, however, suggest that women with cervical cancer have not had a greater tendency to remarry, once their marriage was broken, than other women, and that the primary association may be with the reason for which the marriage was disrupted. Jones *et al.* (1958) found that fewer of the women with cervix cancer had had regular sexual gratification than of the control women, and it is possible that the occurrence of orgasm in some way modifies the susceptibility of the cervical mucosa (Labrum, 1964, personal communication).

SUMMARY

The past experience of 297 patients diagnosed as having cervical cancer has been compared with that of 1353 patients suffering from other diseases. Major factors found to be associated with cancer of the cervix were (i) the married state, (ii) an early age at marriage, and (iii) multiple and broken marriages. Lesser associations were found with (iv) a high frequency of sexual intercourse and (v) lack of use of obstructive methods of contraception. The data clearly support the view that some factor associated with coitus *per se*, rather than with child-bearing, is of primary importance in the causation of the disease.

The following hospitals co-operated in the investigation : Chelsea Hospital for Women, the Central Middlesex Hospital, Hammersmith Hospital, Lambeth Hospital, The London Hospital and the Hospital for Women, Soho Square. We are indebted to the medical staffs for allowing us to interview their patients and to the individual members of the staffs, medical and lay, who notified the cases. We are also indebted to Miss Keena Jones and Miss Rosemary Thomson who interviewed the patients.

### REFERENCES

BRAITHWAITE, J.—(1901) *Lancet*, ii, 1578.
DOLL, R.—(1964) Paper read at a meeting of the International Union against Cancer, Mexico City, February 4, 1964. To be published.
DUNHAM, L. J., THOMAS, L. B., EDGCOMB, J. H. AND STEWART, H. L.—(1960) *Acta Un. int. Cancr.*, **16**, 1689.
DUNN, E. J. AND BUELL, P.—(1959) *J. nat. Cancer Inst.*, **22**, 749.
HAENSZEL, W. AND HILLHOUSE, M.—(1959) *Ibid.*, **22**, 1157.
HEINS, H. C., DENNIS, E. J. AND PRATT-THOMAS, H. R.—(1958) *Amer. J. Obstet. Gynec.*, **76**, 726.
JONES, E. G., MACDONALD, I. AND BRESLOW, L.—(1958) *Ibid.*, **76**, 1.
KENNAWAY, E. L.—(1948) *Brit. J. Cancer*, **2**, 177.
KHANOLKAR, V. R.—(1950) *Acta Un. int. Cancr.*, **6**, 881.
LILLIENFELD, A. M. AND GRAHAM, S.—(1958) *J. nat. Cancer Inst.*, **21**, 713.
LOMBARD, H. L. AND POTTER, E. A.—(1950) *Cancer*, **3**, 960.
MALIPHANT, R. G.—(1949) *Lancet*, i, 978.
PLANT, A. AND KOHN-SPEYER, A. C.—(1947) *Science*, **105**, 391.
PRATT-THOMAS, H. R., HEINS, H. C., LATHAM, E., DENNIS, E. J. AND MCIVER, F. A.—(1956) *Cancer*, **9**, 671.
SHE, M. P., CHENG, F. L., LIU, T. H., T'SAI, H. Y., LIU, C. M. AND WU, E. R.—(1962) ' Cancer Research ', Shanghai (Shanghai Scientific and Technical Publishers).
STERN, E. AND DIXON, W. J.—(1961) *Cancer*, **14**, 153.
STERN, R.—(1842) *Giornali per Servise al Progressi della Pathologia e della Therapeutica*, seria 2, **2**, 507.
STOCKS, P.—(1955) *Brit. J. Cancer*, **9**, 487.
TERRIS, M.—(1962) *Ann. N.Y. Acad. Sci.*, **97**, 808.
*Idem* AND OALMANN, M. C.—(1960) *J. Amer. med. Ass.*, **174**, 1847.
VINEBERG, H. N.—(1906) *Obstet. Gynec.*, **53**, 410.
WYNDER, E. L., CORNFIELD, J., SHROFF, P. D. AND DORAISWAMI, K. R.—(1954) *Amer. J. Obstet. Gynec.*, **68**, 1016.